# Hydrogen-Free Diamond Like Carbon Films with Embedded Cu Nanoparticles: Structure, Composition and Reverse Saturable Absorption Effect

**DOI:** 10.3390/ma13030760

**Published:** 2020-02-07

**Authors:** Šarūnas Meškinis, Andrius Vasiliauskas, Karolis Viskontas, Mindaugas Andrulevičius, Asta Guobienė, Sigitas Tamulevičius

**Affiliations:** 1 Institute of Materials Science, Kaunas University of Technology, K. Baršausko St. 59, LT-51423 Kaunas, Lithuania; andrius.vasiliauskas@ktu.lt (A.V.); mindaugas.andrulevicius@ktu.lt (M.A.); asta.guobiene@ktu.lt (A.G.); sigitas.tamulevicius@ktu.lt (S.T.); 2 JSC Ekspla, Savanorių 237, LT-02300 Vilnius, Lithuania; karolis.viskontas@gmail.com

**Keywords:** diamond like carbon films, nanocomposite, embedded copper nanoparticles, surface plasmon resonance, magnetron sputtering, XPS, Raman spectroscopy, AFM, nonlinear optical properties

## Abstract

In the present research, hydrogen-free diamond like carbon films with embedded copper nanoparticles (DLC:Cu) were grown by simultaneous DC magnetron sputtering of the graphite and copper targets. X-ray photoelectron spectroscopy was used to define the composition of the samples. Atomic force microscopy studies of diamond, like carbon films containing different amount of copper, revealed wide range of the surface morphologies as well as sizes and shapes of the embedded copper nanoclusters. Raman scattering spectra of all the DLC:Cu films investigated were typical for diamond-like carbon (including samples containing more than 60 at.% of copper). sp^3^/sp^2^ carbon bond ratio in the films decreased with the increase of the Cu amount in the films. According to the optical absorbance measurements, the surface plasmon resonance related absorption peak of DLC:Cu films was only detected in the films containing ≥28.45 at.% Cu. For the diamond like carbon films containing more than 40 at.% Cu, a further increase of Cu amount in the nanocomposite resulted in minor changes of the absorbance spectra. Some correlation between the changes of the samples surface morphology as well as phase structure and optical absorbance spectra of the films was found. In all cases, reverse-saturable absorption of the DLC:Cu films was observed. For some DLC:Cu films damage of the sample occurred at higher light fluences that can be related to the heating that is caused by the surface plasmon resonance effect.

## 1. Introduction

Diamond like carbon (DLC) is an umbrella name that is used for a wide group of amorphous carbon materials. DLC consists of carbon atoms bonded by sp^2^ (like in graphite) and sp^3^ (like in diamond) bonds [1,2,3]. In addition to that, DLC can contain 0–40 at.% of hydrogen [1,2,3]. The properties of these films can be additionally controlled by growing diamond like carbon films containing chemical elements other than carbon or hydrogen [1,3]. Diamond like carbon films can be deposited at room temperature while using plasma based methods [1,2,3]. DLC films are hard, wear and corrosion resistant, and biocompatible [1,2,3,4,5,6]. Their optical transmittance and refractive index can be tuned in a wide range [1,2,3]. Recently, nonlinear optical properties of the diamond like carbon films were considered. Particularly, it was supposed that embedding diamond-like carbon particles to the film used for nonlinear optical applications might improve the saturable absorption effect (i.e., larger increase of a light transmittance with increased light beam intensity) [7]. Saturable and reverse saturable absorption phenomena were both observed in hydrogenated diamond like carbon films with embedded Cu nanoparticles (DLC:Cu) [8]. DLC films became a very interesting alternative for presently used nonlinear optical materials, such as semiconductor self saturable absorber mirrors [9], plasmonic nanoparticles of group IB metals (Au, Ag or Cu) [10,11], graphene [9], or carbon nanotubes [12], due to the beneficial combination of the mechanical and optical properties mentioned above as well as room temperature deposition possibility. However, as mentioned above, there are only few studies on diamond like carbon as a nonlinear optical material [8].

Taking the interesting nonlinear optical properties of the hydrogenated DLC:Cu films mentioned above into account, as well as the significant effects of the hydrogen on properties of the diamond like carbon [1,3], in the present research hydrogen-free DLC films containing copper were studied to reveal possible relations between the composition, structure, and nonlinear optical properties of the films.

## 2. Materials and Methods

The DLC:Cu films were deposited at room temperature by simultaneous direct current (DC) magnetron sputtering of graphite and copper targets. The diameters of both targets were 3″. Ar was used as a sputtering gas. Residual gas pressure and work pressure in the deposition chamber was 6 × 10^−4^ Pa and (7 ± 1) × 10^−1^ Pa, respectively. The substrate–target gap was 0.1 m. The substrates were grounded. Before deposition, all of the substrates were washed by subsequent boiling in dimethylformamide and acetone to remove organic contaminants. Cleaning of the substrates was completed by immersing them into the flowing deionized water. Table 1 presents other deposition details.

The DLC:Cu films that were deposited on Si(100) substrates were used for X-ray photoelectron spectroscopy and Raman scattering spectroscopy measurements. The samples for study of the linear and nonlinear optical properties were grown on fused silica substrates. The thickness of the studied films was in the range of 10–40 nm.

Raman microscope inVia (Renishaw, Wotton-under-Edge, UK) with 532 nm wavelength excitation (excitation power 1.5 mW) was employed in Raman scattering measurements. The integration time was set at 100 s. The grating with groove density of 2400 grooves/mm was used. Two Gaussian shape components (G and D peaks) were used to determine the parameters of the Raman scattering spectra. The fitting of the experimental curves was completed using X-ray photoelectron spectra (XPS) peak v3.1 software (free software written by Ramund Kwok). Thus, conventional fitting procedure was used. D and G peak area ratio (D/G area ratio), G peak position, and G peak Full Width at Half Maximum (FWHM(G)) were calculated as the parameters that are proportional to the sp^3^/sp^2^ carbon ratio in the film [1,13].

The X-ray photoelectron spectra (XPS) of the samples were acquired by a Thermo Scientific ESCALAB 250Xi spectrometer (Thermo Fisher Scientific, East Grinstead, UK) while using a monochromatic Al Kα radiation (hν = 1486.6 eV). The survey and high-resolution spectra were recorded using the 40 and 20 eV pass energy values of a hemispherical electron energy analyser, respectively. Au 4f_7/2_, Ag 3d_5/2_, and Cu 2p_3/2_ peak positions were used for the calibration of the energy scale of the system. Data analysis was executed by using the Thermo Scientific Advantage software (v5.979).

An optical spectrometer Avantes composed of a deuterium halogen light source (AvaLight DHc, Avantes, Apeldoorn, The Netherlands) and spectrometer (Avaspec-2048, Avantes, Apeldoorn, The Netherlands) was used to study the linear optical properties of the films. The absorbance of the films was analysed in the wavelength region from 200 nm to 1000 nm (possible optical losses related to reflectance were not taken into account).

The nonlinear optical response of the selected samples was acquired by a nonlinear reflectance measurement system. Picosecond mode locked fiber laser (JSC Ekspla, Vilnius, Lithuania) was applied as a variable wavelength light source (1020–1080 nm range). The samples were put on a dielectric Bragg mirror and excited by a pulsed laser beam. The wavelength of the exciting laser beam was 1064 nm. The laser beam diameter was 6.6 µm. Pulse time used has been ~8 ps. Excitation fluence up to 1 mJ/cm^2^ was used. It corresponds to a highest applied optical power of 0.125 GW/cm^2^. The nonlinear reflectance was also acquired at 1064 nm. More details on the measurement setup used may found elsewhere [8,14].

## 3. Results

The X-ray photoelectron spectra of diamond like carbon films with embedded nanoparticles were acquired before and after the sample’s surface cleaning by Ar^+^ ion beam.

**Figure 1:**
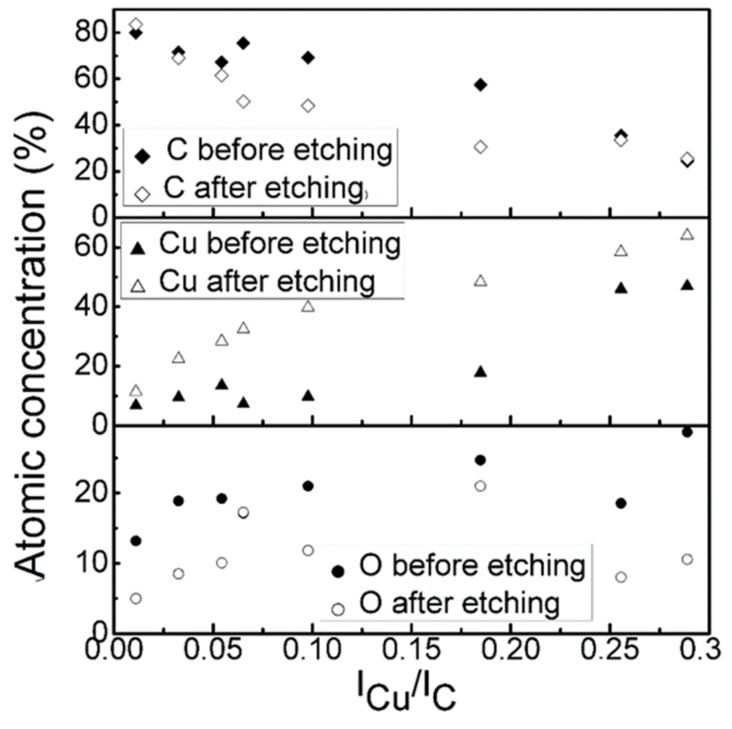
Chemical composition of DLC:Cu films Vs copper and carbon target current ratio.

**Figure 2:**
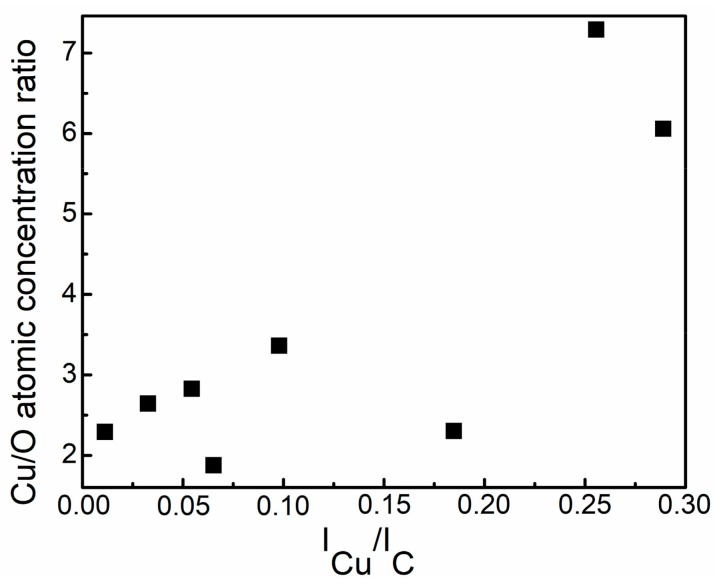
Copper and oxygen (Cu/O) atomic concentration ratio Vs copper and carbon target current ratio (I_Cu_/I_C_). Atomic concentrations measured after the *In situ* ion beam etching were analysed.

Figure 1 presents the dependence of the chemical composition of the DLC:Cu films on copper and carbon target current ratio. It should be noted that the copper content on the surface significantly increased, after the applied sputtering procedure (curves noted as “after etching”). Analysis of the DLC:Cu films cleaned by Ar^+^ ion beam revealed quasilinear increase of the Cu atomic concentration with the copper target sputtering current (copper and graphite targets current ratio) used. The copper amount increased with copper and carbon target current ratio. The oxygen atomic concentration non-monotonically varied with Cu and C magnetron current ratio, after initial increase, for the DLC:Cu films containing the largest amount of copper, the oxygen atomic concentration decreased. On the other hand, the tendency of Cu/O atomic concentration ratio to increase with the Cu and C target current ratio was found (Figure 2). Oxygen amount in the deposited films varied in 5–18 at. % range.

**Figure 3:**
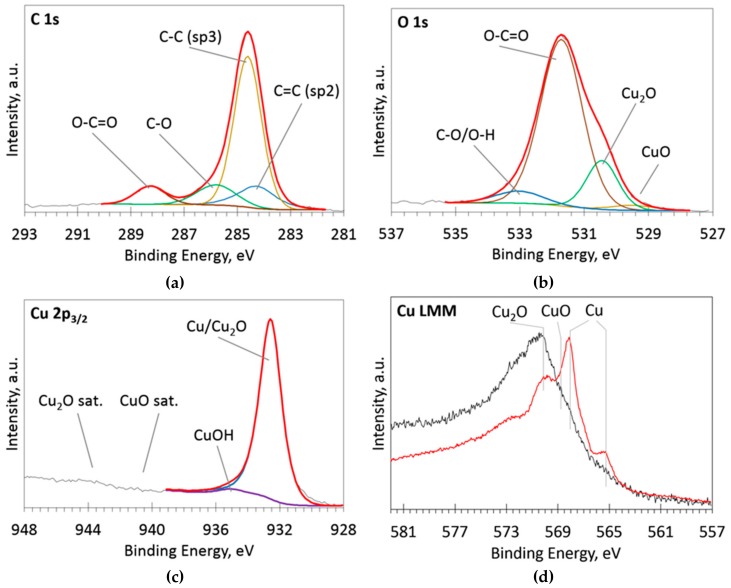
Deconvoluted high resolution C1s (**a**), O1s (**b**), Cu2p (**c**), Cu LMM (**d**) X-ray photoelectron spectra (XPS) spectra of the sample Cu09. Black thin line–acquired data, colored thick lines—fitted peaks, red thick line—envelope.

Analyzing C1s, O1s, Cu2p, and Cu LMM XPS peaks was undertaken to investigate chemical bonds on the surface of the ion beam cleaned samples. The typical spectra are presented in Figure 3. C-C, C=C, C-O, and O-C=O bonds [15] were revealed by analysis of C1s peak (Figure 3a). Cu_2_O fitting component is present in O1s peak [16,17], while the CuO fitting component is rather weak. Only Cu and Cu_2_O peaks [16,18] were found in the Cu2p and Cu LMM spectra. No CuO peak was observed. Thus, one can suppose that copper nanoparticles that are embedded into the DLC matrix consist of copper and Cu_2_O.

**Figure 4:**
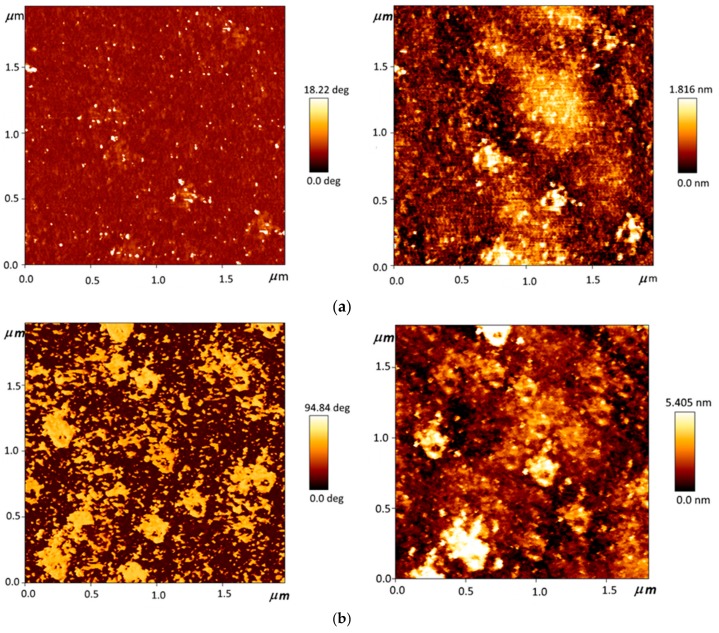

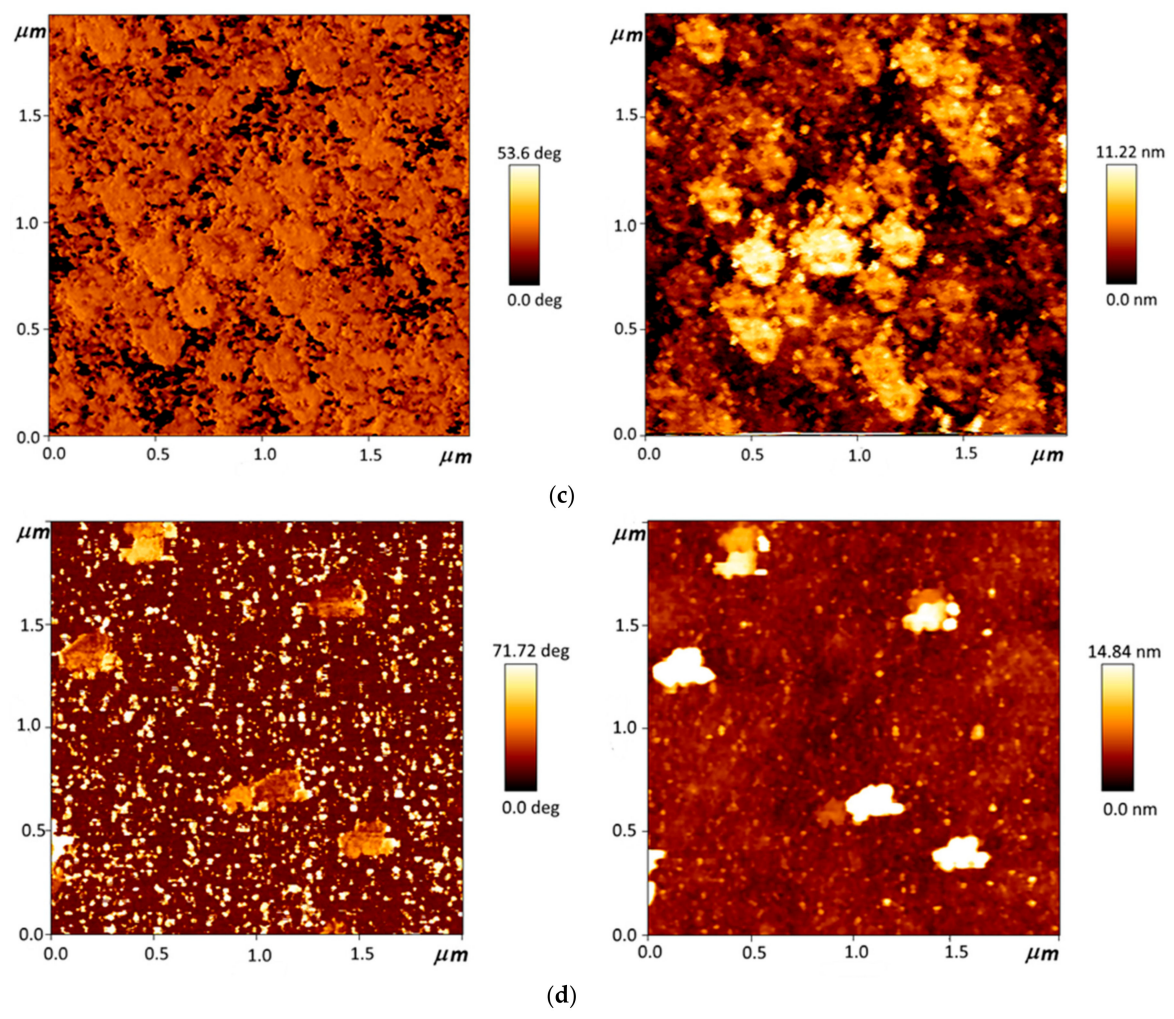
Atomic force microscopy (AFM) images of DLC:Cu samples containing different amount of copper: 22.48 at.% Cu (**a**), 32.45 at.% Cu (**b**), 48.36 at.% Cu (**c**), 58.47 at.% Cu (**d**). Phase images are presented at the left and morphology images are at the right.

**Figure 5:**
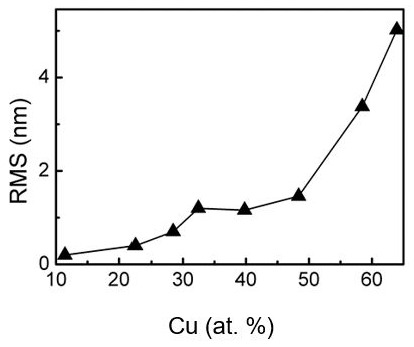
RMS of DLC:Cu films Vs Cu amount.

Figure 4 presents the atomic force microscopy (AFM) images of DLC:Cu samples containing different amount of copper. One can see that the surface morphology of DLC:Cu film varies with Cu atomic concentration. At the first (in the low Cu concentration region), it is ultrasmooth (RMS = 196 pm) (see Supplementary Materials Figure S1a). However, some solitary point-like structures can be seen in the phase image (Figure S1a). The increased Cu atomic concentration resulted in an agglomeration of the point structures to the aggregates (nanoclusters) (Figure 4a) and a slight increase of RMS of the films (Figure 5). Further increase of copper amount was followed by the increased number of clusters (Figure S1b). Afterwards, gaps between the nanoclusters were filled by point-like structures, resulting in an increase of RMS (Figure 4b). Finally, the stable state was reached, despite further increase in Cu atomic concentration (Figure S1c and Figure 4c). Secondary point structures and nanoclusters formation can explain this surface morphology evolution. When the Cu amount in DLC:Cu film exceeds 50 at.%, nanoclusters begin to grow vertically (Figure 4d and Figure S1d). As a result, RMS significantly increased, up to 5 nm (Figure 5). Thus, a wide range of the surface morphologies as well as sizes and shapes of the embedded copper nanoclusters were found in DLC:Cu films containing different amount of copper (Figure 4 and Figure S1).

It should be noted that the RMS of DLC:Cu films containing up to 23 at.% copper was close to the roughness that was reported for undoped DLC films [1,2]. However, roughness increased with the Cu atomic concentration and the RMS values for DLC:Cu films containing >50 at.% of copper were significantly higher.

**Figure 6:**
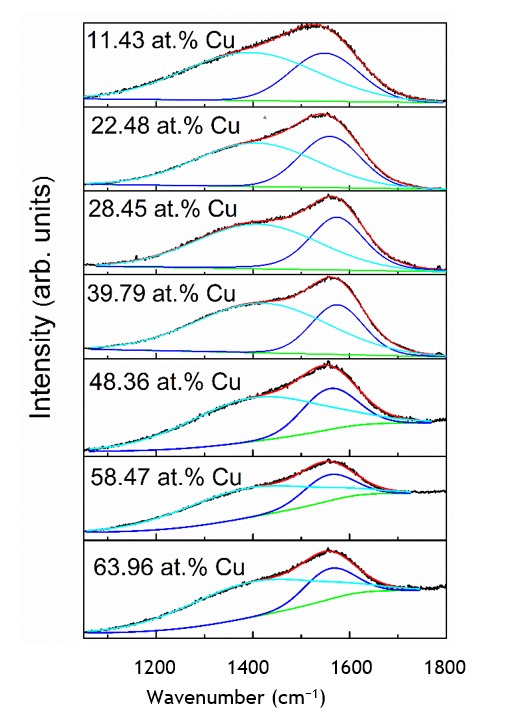
The representative Raman scattering spectra of hydrogen-free DLC:Cu films.

Figure 6 illustrates the representative Raman scattering spectra of the diamond like carbon films with embedded Cu nanoparticles. All of the spectra are typical for diamond like carbon [1]. A wide spectral feature in the 1200–1700 cm^−1^ wavenumber range can be seen. That feature is usually defined as a superposition of two peaks. The main peak at ~1400–1600 cm^−1^ is explained as a stretching vibration mode of sp^2^-bonded carbon atoms. It is called G peak. While a shoulder in ~1200–1400 cm^−1^ wavenumbers range is explained as a disorder-induced D peak that is associated with the sp^2^-bonded carbon ring breathing mode [1]. The shape of the spectra in all cases was rather similar, despite Cu atomic concentration in the films varying in 10–60 at.% range and the carbon content in some samples being as low as 25 at.%. It must be noted that such a result is in accordance with the recent findings. In [8], the Raman scattering spectra of the hydrogenated amorphous carbon and copper nanocomposites were typical for DLC, even in the case when carbon atomic concentration in the films was 20 at.%. In [19], Raman scattering spectra that are typical for DLC were reported for amorphous carbon films containing 50 at.% of copper. In [20], the shape of the Raman scattering spectra of the hydrogenated DLC:Cu films was nearly the same for the samples containing 27.85 at.% and 77.31 at.% Cu.

It should be mentioned that, for the samples containing >40 at.% of copper, the luminescence background appeared in the Raman scattering spectra. The luminescence background was reported for Raman spectra of the as-grown graphene sample, directly acquired on the copper foil [21]. Photoluminescence was also reported for copper nanoparticles [22]. Thus, it can be supposed that the luminescence background seen in the Raman scattering spectra of DLC:Cu films is due to the surface plasmon resonance related luminescence of embedded copper nanoparticles.

**Figure 7:**
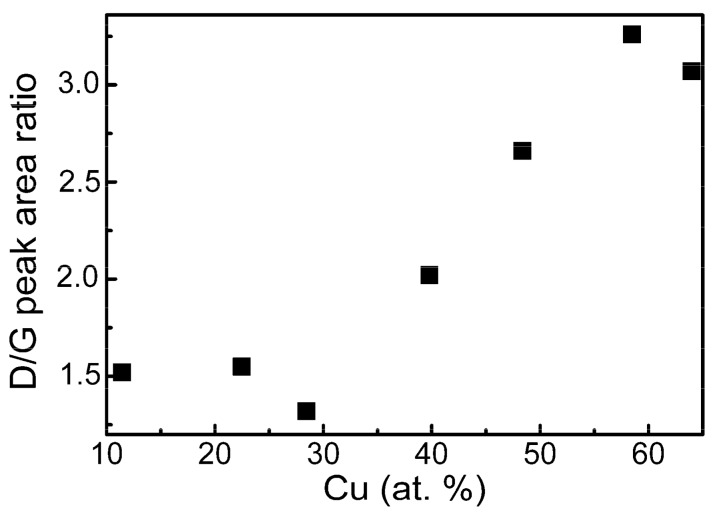
D/G peak area ratio Vs copper atomic concentration in DLC:Cu films.

Figure 7 presents the Raman scattering spectra parameters and their dependence on the chemical composition of DLC:Cu films. It can be seen that D/G peak area ratio increases with the increase of Cu atomic concentration in the nanocomposite film. According to [1,13], it means that the sp^3^/sp^2^ carbon bond ratio decreases with the increase of Cu amount in the films. It should be mentioned that similar behaviour was found for the DLC:Cu films in other studies (please, see review [5], and references therein).

**Figure 8:**
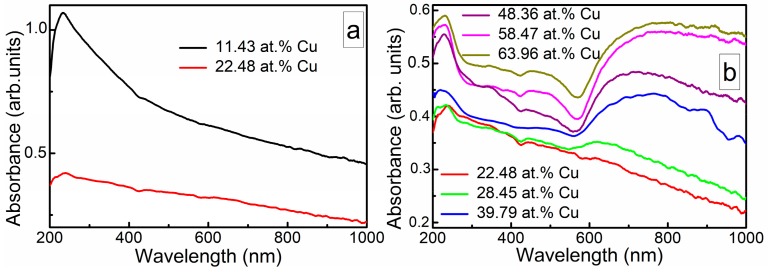
The representative optical absorbance spectra of DLC:Cu films: ≤22.48 at.% Cu, disappearance of diamond like carbon (DLC) related absorption peak at lower wavelengths (**a**); ≥22.48 at.% Cu, rise of the plasmonic peak (**b**). Absolute absorbance values are adjusted to make their analysis easier. The absorbance curves were lined up according to the Cu amount in the films in (**b**).

Figure 8 presents the tptical absorbance spectra of DLC:Cu films. The absorbance spectra of DLC:Cu films containing 11.43 at.% Cu are similar to the absorbance spectra of conventional undoped DLC films [23,24,25,26], i.e., absorbance maximum is seen in near ultraviolet range (at ~240 nm) and the absorbance decreases with the wavelength. The increase in Cu amount up to 22.48 at.% results in a substantial decrease of the light absorbance in the lower wavelengths range and absorption maximum mentioned above in this study is barely seen. The increase of copper atomic concentration up to 28.45 at.% results in the appearance of the low intensity broad plasmonic peak with a maximum at ~620 nm wavelength. The plasmonic absorbance peak is related to the formation of copper nanoparticles embedded in the amorphous carbon matrix. Significantly increased surface roughness and phase contrast (Figure 4 and Figure 5) supports this assumption. A further increase of Cu amount up to 40 at.% results in redshift and substantially increased the intensity of the plasmonic peak. A further increase of Cu amount in the nanocomposite films resulted in some increase of the broad plasmonic peak intensity. No clear shift of the plasmonic peak position with a copper amount in the films was seen. Table 2 presents some correlation between the changes of the DLC:Cu films surface morphology as well as phase structure and optical absorbance spectra of the films.

It should be mentioned that, in the case of the hydrogenated DLC:Cu films, traces of the plasmonic absorbance peak were observed for the samples containing 15–17 at.% of Cu [26]. For the hydrogenated DLC:Cu films containing 22 at.% of copper, plasmonic peak was clearly seen. In addition, plasmonic absorbance peaks of the hydrogen-free DLC:Cu films are broader and redshifted in comparison with the hydrogenated ones (data on hydrogenated DLC:Cu films, please see in [8,26]).

**Figure 9:**
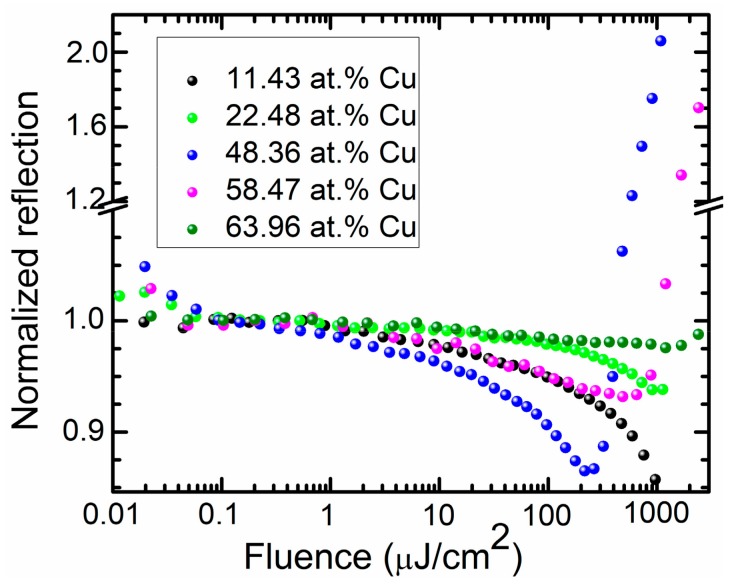
The normalized reflectance vs laser fluence for the selected hydrogen-free DLC:Cu films.

Figure 9 illustrates the typical dependencies of the normalized reflectance of selected samples on laser fluence. Reverse-saturable absorption (reflectance decrease with laser fluence) was found for the DLC:Cu films containing lower quantities of copper (11.43 and 22.48 at. %). In the case of the sample containing 48.36 at.% Cu, a reverse saturable absorption effect was observed for fluences lower than 200 μJ/cm^2^. Afterwards, damage of the sample took place. The observed increase of the light transmittance as a result of higher fluence laser irradiation was irreversible. Similar behaviour was observed for the DLC:Cu sample containing 58.47 at.% Cu. Yet, in this case, a decrease of the normalized reflectance was smaller and sample damage was observed at higher light fluences. In the case of the DLC:Cu film containing 63.96 at.% Cu, a decrease of the normalized reflectance was rather small (less than 2%). In this case, the sample was damaged at even higher light fluence of ~2000 J/cm^2^. It seems that laser beam induced damage in some DLC:Cu films is related to the surface plasmon resonance effects. For the DLC:Cu film containing 48.36 at.% Cu relatively low fluence light induced damage effect correlates with significantly increased intensity of plasmonic absorption peak and the appearance of the Raman scattering spectra luminescence background. In such a case, plasmonic heating should be taken into account, i.e. temperature increase due to the selective absorption of light by plasmonic nanostructures [27]. It is known that annealing has significant effects on the structure of DLC films [1,28,29,30]. However, graphitization of the diamond like carbon usually takes place as a result of the annealing [1,28,29,30], which results in decreased light transmittance. From such a point of view, an increase of DLC:Cu films optical transmittance as a result of the laser induced damage appears to be surprising. Thus, the possible changes of the copper phase, such as air annealing induced oxidation, should be taken into account. Particularly, in [31] annealing of DLC:Cu film in Ar gas ambient at 300 °C temperature resulted in the formation of Cu_2_O phase due to the residual gas effects. Thus, in our case, such effects could be substantially more pronounced.

In the present study, no saturable absorption effects were observed in hydrogen-free DLC:Cu films. Only optical limiting effect (i.e., increased absorbance with increased light intensity) was found. That is the behaviour different from hydrogenated DLC:Cu films [8]. In that case, a saturable absorption effect was seen for the films containing ≥58 at.% Cu. No correlation between the plasmonic optical absorption peak and saturable absorption effect was found for the hydrogenated DLC:Cu films. As discussed above, the oxidation of the embedded Cu nanoparticles should be taken into account [32]. Nevertheless, in our case, it can not explain the observed differences between the nonlinear optical properties of hydrogenated and hydrogen-free DLC:Cu films. The analysis of oxygen amount and Cu/O atomic concentration ratio for both groups of the films did not reveal any differences, which could explain differences of the nonlinear optical properties that are mentioned above (please see present study and [8]). Thus, differences may be related to the presence/absence of hydrogen in amorphous carbon matrix.

## 4. Conclusions

In conclusion, hydrogen free DLC:Cu nanocomposite films that were grown by simultaneous DC magnetron of graphite and copper targets were studied. The investigation of chemical composition of the deposited films revealed that Cu atomic concentration and Cu/O atomic concentration ratio increased with the copper and graphite targets current ratio used. A wide range of the surface morphologies, as well as sizes and shapes of the embedded copper nanoclusters, were found in DLC:Cu films containing different amount of copper. Raman scattering spectra of all studied samples were typical for diamond like carbon, even for the DLC:Cu films containing more than 60 at.% of copper. However, for the samples containing >30 at.% of copper, luminescence background appeared in the Raman scattering spectra. It was explained by the surface plasmon resonance related luminescence of embedded copper nanoparticles. sp^3^/sp^2^ carbon bond ratio was dependent on copper concentration and decreased with the increased Cu amount in the films, according to the Raman scattering spectra parameters. Study of the optical absorbance of DLC:Cu films revealed that the low intensity broad plasmonic peak with maximum at ~620 nm wavelength only appears with the increase of copper amount up to 28.45 at.%. The increase of Cu amount up to 40 at.% resulted in redshift and substantially increased the intensity of the plasmonic peak. A further increase of the Cu amount in the nanocomposite films resulted in minor changes of the absorbance spectra. Some correlation between the changes of the samples surface morphology, as well as phase structure and optical absorbance spectra of the films, was found. Reverse-saturable absorption was observed for all of the samples. The smallest decrease of the reflectance (less than 2%) was found for the DLC:Cu film containing 63.96 at.% Cu. For the DLC:Cu films containing 48.36 at.% and 58.47 at.% of copper, optical damage of the sample occurred at higher light fluences. It seems that this laser beam induced damage can be related to heating that is caused by the surface plasmon resonance effect.

## Figures and Tables

**Table 1:** Numbering of the samples and other deposition conditions used in magnetron sputtering deposition of DLC:Cu films.

Sample No	Current (A)		Ar Gas Flow (sccm)		Voltage (V)	
	Cu Target	C Target	Cu Target	C Target	Cu Target	C Target
Cu01	0.01	0.9	56	49	285	732
Cu03	0.03	0.9	56	49	295	729
Cu05	0.05	0.91	56	49	299	723
Cu06	0.06	0.92	56	49	303	722
Cu09	0.09	0.92	56	49	309	718
Cu17	0.17	0.9	56	49	324	728
Cu25	0.25	0.9	56	49	330	704
Cu35	0.35	0.9	56	49	331	705

**Table 2:** Correlation between the changes of the DLC:Cu films surface morphology as well as phase structure and the optical absorbance spectra of the films.

Surface Morphology and Phase Structure According to AFM	Main Features of Optical Absorbance Spectra
Ultrasmooth DLC:Cu film (RMS = 196 pm). Some solitary point-like structures can be seen in phase image	Absorption spectra typical for undoped DLC.
Agglomeration of the point structures to the aggregates. RMS of the films slightly increase.	Significant decrease of the light absorbance in lower wavelengths range.
Increased number of the observed clusters. Significant increase of the RMS (5 times).	Appearance of the weak plasmonic peak.
Secondary point structures and nanoclusters formation. No further increase of the RMS.	Plasmonic peak redshift and significant increase of the intensity.
Nanoclusters began to grow vertically and RMS increase again.	Only slight changes of the absorbance spectra.
Ultrasmooth DLC:Cu film (RMS = 196 pm). Some solitary point-like structures can be seen in phase image.	Absorption spectra typical for undoped DLC.
Agglomeration of the point structures to the aggregates. RMS of the films slightly increase.	Significant decrease of the light absorbance in lower wavelengths range.

## Supplementary Materials

The following are available online at https://www.mdpi.com/1996-1944/13/3/760/s1, Figure S1: AFM images of DLC:Cu samples containing different amount of copper.
