# Supplementary material for: Hydrogen-Free Diamond Like Carbon Films with Embedded Cu Nanoparticles: Structure, Composition and Reverse Saturable Absorption Effect
Source: Materials (Basel). 2020 Feb 7;13(3):760. doi: 10.3390/ma13030760 (PMC7041493; doi:10.3390/ma13030760)
Supplement: Supplementary file 1 [file materials-13-00760-s001.pdf]

materials

Supplementary Materials

Hydrogen-Free Diamond Like Carbon Films with Embedded Cu Nanoparticles: Structure, Composition and Reverse Saturable Absorption Effect

Šarūnas Meškinis [1,]*, Andrius Vasiliauskas [1], Karolis Viskontas [2], Mindaugas Andrulevičius [1], Asta Guobienė [1], and Sigitas Tamulevičius [1]

[1] Institute of Materials Science, Kaunas University of Technology, K. Baršausko St. 59, LT-51423 Kaunas, Lithuania; andrius.vasiliauskas@ktu.lt (A.V.); mindaugas.andrulevicius@ktu.lt (M.A.); asta.guobiene@ktu.lt (A.G.); sigitas.tamulevicius@ktu.lt (S.T.)

[2] JSC Ekspla, Savanorių 237, LT-02300 Vilnius, Lithuania; karolis.viskontas@gmail.com

* Correspondence: sarunas.meskinis@ktu.lt

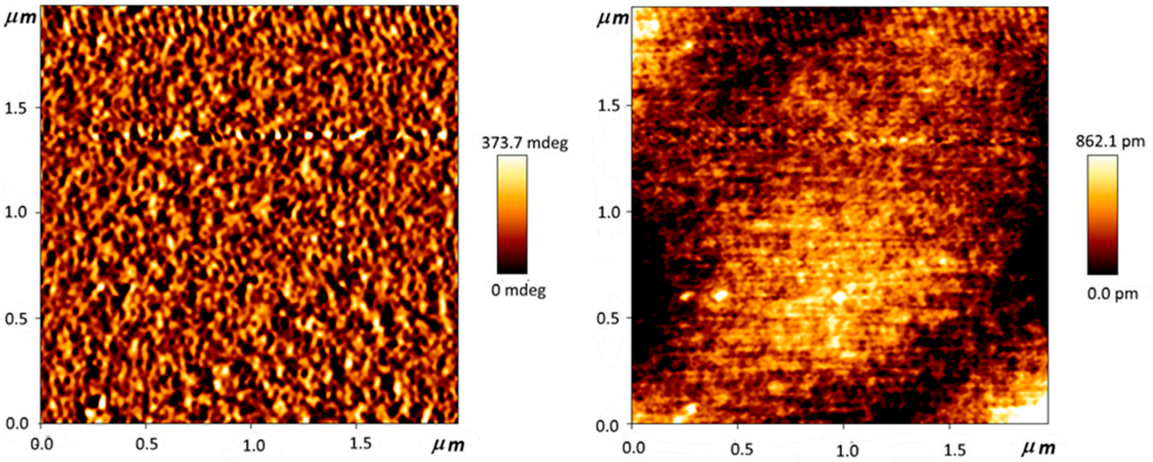

(**a**)

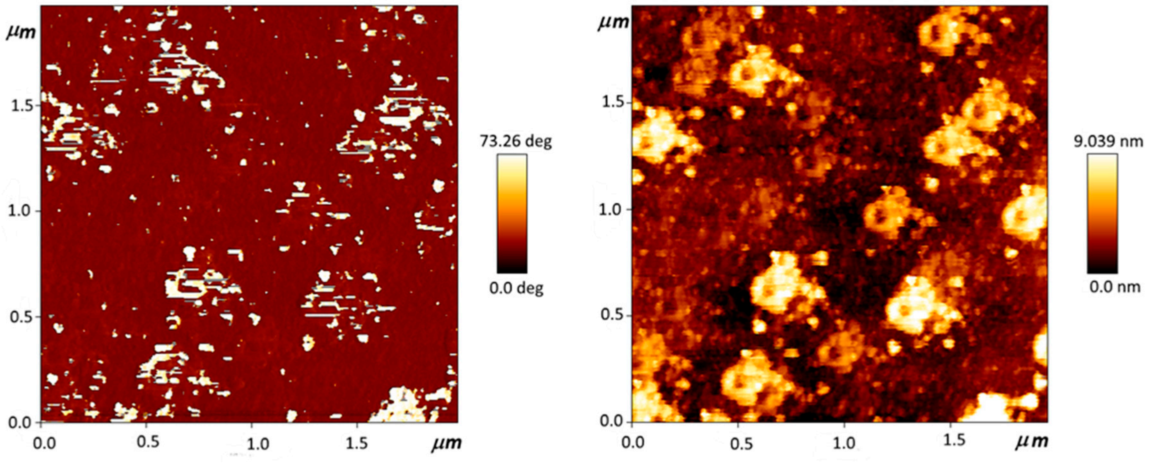

(**b**)

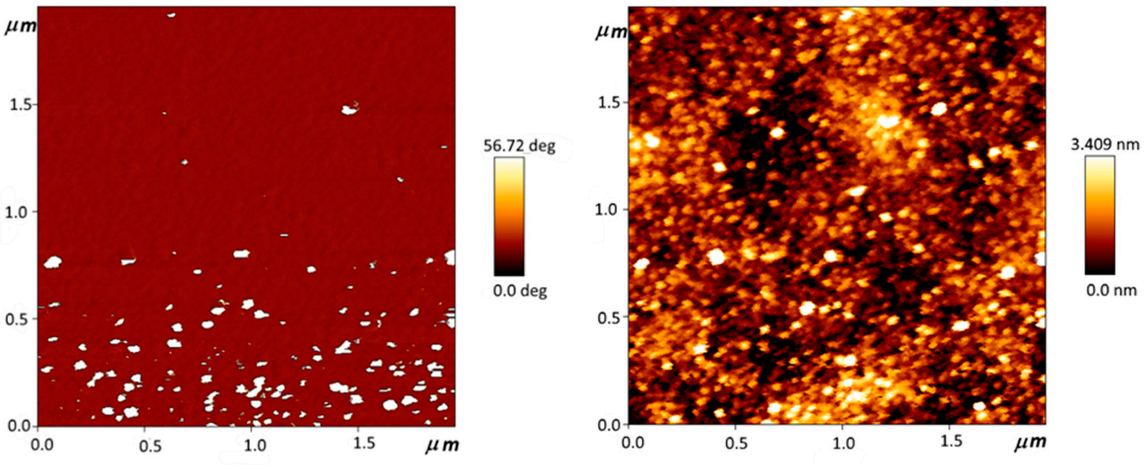

(**c**)

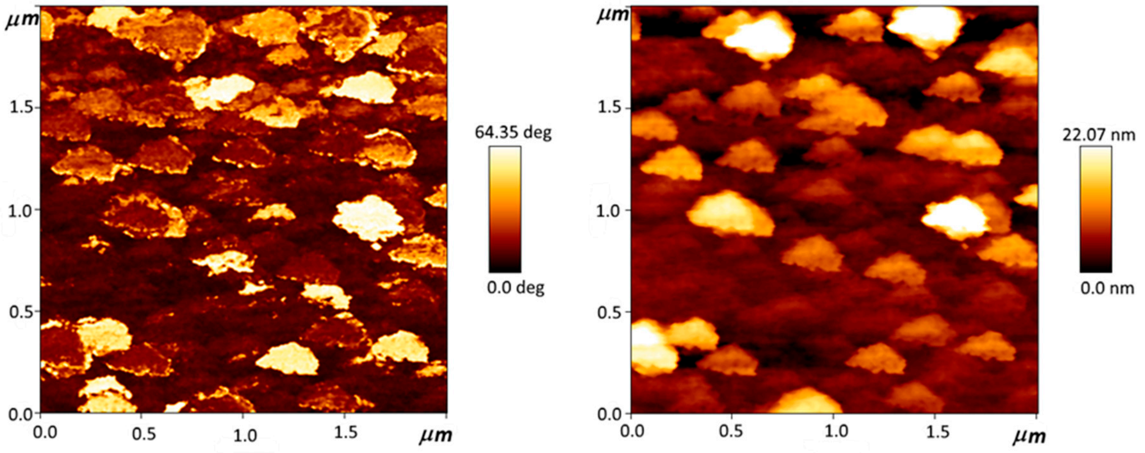

(**d**)

Figure S1. Atomic force microscopy (AFM) images of DLC:Cu samples containing different amount of copper: 11.43 at.% Cu **(a)**, 28.45 at.% Cu **(b)**, 39.79 at.% Cu **(c)**, 63.96 at.% Cu **(d)**. Phase images are at the left and morphology images are at the right.

© 2020 by the authors. Submitted for possible open access publication under the terms and conditions of the Creative Commons Attribution (CC BY) license (http://creativecommons.org/licenses/by/4.0/).
